# Special Issue “The 25th Anniversary of NO”

**DOI:** 10.3390/ijms26136058

**Published:** 2025-06-24

**Authors:** Daniele Mancardi, Michele Samaja

**Affiliations:** 1 Department of Clinical and Biological Sciences, University of Torino, San Luigi Gonzaga Hospital Regione Gonzole 10, 10043 Orbassano, Italy; 2 Department of Health Science, University of Milan, 20142 Milan, Italy

Nitric oxide (NO), a simple yet remarkably versatile molecule, stands today as a pivotal player in virtually all body systems and cellular compartments [1]. NO, first identified by Joseph Priestley in 1772 [2], was originally recognized as one of the many components of gasses derived from the exhaust of industrial plants and was labelled as toxic because of its ability to form strong bonds to haem-proteins [3]. Moreover, NO is a free radical that should be mentioned as •NO because it contains an unpaired electron, which makes it highly reactive and capable of participating in chemical reactions with other molecules [4]. Furthermore, NO can rapidly react with oxygen to form nitrogen dioxide, a potent pulmonary irritant [5]. However, at sub-micromolar levels, NO is not only non-toxic but also carries a number of protective functions in the body. First, but not less important, NO is the major candidate as the endothelium-derived relaxing factor (EDRF) [6,7].

The discovery of at least three isoforms of NO synthase (NOS)—the enzyme family responsible for endogenous NO production—unlocked our understanding of NO’s roles across a wide range of biological contexts. These isoforms include neuronal NOS (NOS1) identified in neuronal tissues [8], inducible NOS (NOS2) described in macrophages [9], and endothelial NOS (NOS3) found in endothelial cells [10]. Notably, these isoforms differ not only in their tissue distribution but also in their physicochemical properties, such as substrate affinity and maximal catalytic activity [11]. These differences allow each NOS isoform to respond uniquely to environmental stimuli, underscoring their functional versatility.

Over 285,000 publications related to NO were recorded between 1815 and 2022, with more than 220,000 of these in the last 20 years alone. NO was proclaimed the “Molecule of the year” in 1992 [12]. Although the biological significance of NO was recognized in the 1990s, particularly through the pioneering work of Salvador Moncada [13], it was the awarding of the 1998 Nobel Prize in Physiology or Medicine to Robert F. Furchgott, Louis J. Ignarro, and Ferid Murad “for their discoveries concerning nitric oxide as a signaling molecule in the cardiovascular system” that cemented NO’s place in biomedical science (https://www.nobelprize.org/prizes/medicine/1998/summary/(accessed on 11 June 2025)).

This Special Issue commemorates the 25th anniversary of that milestone, bringing together contributions from leading scientists worldwide. The aim is to highlight the persistent scientific attention to the pleiotropic roles of NO across physiological systems and its relevance as a therapeutic target—paving the way for similar gaseous mediators such as carbon monoxide [14] and hydrogen sulphide [15].

Ito et al. (2023) [16] examined the impact of aging and hypertension on NO production during cerebral ischemia and reperfusion in wild-type and spontaneously hypertensive rats. The in vivo microdialysis data revealed that aging significantly impairs NO production, particularly in hypertensive models, emphasizing the role of NO in neurovascular aging and ischemic injury.

Poeggeler et al. (2023) [17] discussed how supplementation with L-arginine and B vitamins enhances NO synthesis, mitigates oxidative stress, and supports healthy aging. Their review delves into the biochemical interplay between NO bioavailability and reactive oxygen species, particularly superoxide anions, highlighting implications for cardiovascular health and clinical trials underway.

Samaja et al. (2023) [18] reflected on why haemoglobin-based oxygen carriers (HBOCs) have failed clinical translation over the past 50 years. A major obstacle remains NO scavenging by acellular haemoglobin, leading to vasoconstriction, inflammation, and coagulopathies. Their work underscores the need for molecular strategies to mitigate NO reactivity in blood substitute development.

Paterno and colleagues (2023) [19] demonstrated that nasal NO (nNO) levels serve as a valuable diagnostic biomarker for primary ciliary dyskinesia (PCD). Their literature review established reliable nNO cut-off values and emphasized NO’s broader implications in respiratory health and disease phenotyping.

Coutinho et al. (2024) [20] explored the co-expression of inducible NOS (NOS2) and inducible cyclooxygenase (COX-2) in the tumour microenvironment, showing strong associations with poor survival in aggressive cancers, particularly oestrogen receptor-negative breast cancer. Their work provides insights into spatial expression patterns and suggests targeted therapeutic approaches involving NO and inflammatory pathways.

Scarpellino et al. (2024) [21] challenged the neurocentric view of NO signalling by demonstrating a key role for endothelial NOS (NOS3) in neurovascular coupling and synaptic plasticity. Their review advocates for a broader perspective on how endothelial cells and cerebrovascular responses integrate neural and vascular signals.

Taken together, these articles emphasize the role of NO as a quintessential pleiotropic molecule. Indeed, few molecules, if any, exhibit the biological versatility of NO. Its pleiotropic effects, not all of which are covered in this Special Issue, span virtually all physiological systems, including:

- Cardiovascular system: vasodilation, blood pressure regulation, and anti-atherosclerotic activity.
- Nervous system: neurotransmission, neurovascular coupling, and synaptic plasticity.
- Immune system: antimicrobial and anti-tumour functions via macrophage activation.
- Coagulation system: inhibition of platelet aggregation and thrombus formation.
- Respiratory system: regulation of bronchial tone and gas exchange.
- Reproductive system: smooth muscle relaxation and erectile function.
- Renal system: regulation of glomerular filtration and renal blood flow.
- Gastrointestinal system: control of motility and sphincter relaxation.
- Mitochondrial function: modulation of oxidative phosphorylation and cellular respiration.
- Hypoxia and ischemia response: adaptation to oxygen deprivation at systemic and cellular levels.

Owing to the multifaceted roles of NO described above, this molecule is directly or indirectly implied in myriad clinical applications:

- Inhaled NO is employed in persistent pulmonary hypertension of the newborn, cardiac surgery and transplantation, acute respiratory distress syndrome, severe hypoxemia, and sickle cell disease. Administered as a gas, NO exerts vasodilatory effects within the pulmonary circulation without inducing systemic hypotension, making its use valuable in critical care and pulmonary medicine.
- The infusion of L-arginine, the substrate of NOS, increases overall NO bioavailability with benefits in pulmonary hypertension, peripheral artery disease, ischemic heart disease, hypercoagulopathies, erectile dysfunction, and inflammatory states, also providing metabolic and renal support by improving glucose metabolism in insulin-resistant states and insulin sensitivity.
- NO donors (i.e., nitroglycerin, S-nitrosothiols, NONOates, sodium nitroprusside, furoxans), compounds that release NO in the circulation, are used for their vasodilatory, anti-aggregatory, and cytoprotective properties in angina pectoris, heart failure, peripheral vascular disease, and erectile dysfunction.
- Phosphodiesterase (PDE) inhibitors block the degradation of the cyclic nucleotides cAMP and cGMP, increasing NO-driven downstream effects like vasodilation, cardiac stimulation, bronchodilation, and anti-inflammatory responses. Up to eleven PDE inhibitor classes are known, localized in various body districts as the cardiovascular, pulmonary, erectile, autoimmune and inflammatory systems.
- Guanylate cyclase stimulators and agonists stimulate guanylate cyclase enzymes, increasing intracellular cGMP bypassing or enhancing the NO pathway, with benefits in conditions where endogenous NO production is impaired, such as pulmonary hypertension (Riociguat), heart failure (Vericiguat), and gastrointestinal disorders (Linaclotide).

In conclusion, this Special Issue pays tribute to NO as a molecule of immense scientific and clinical significance. Its discovery not only transformed our understanding of vascular biology but also opened new avenues in neuroscience, immunology, oncology, and beyond. As we celebrate the 25th anniversary of the Nobel Prize that acknowledged NO’s pivotal role, the collective contributions in this issue underscore the molecule’s enduring impact and its promise for future therapeutic innovation, with a great deal of investigations still ongoing. Nitric oxide: a pleiotropic molecule at the crossroads of physiology and pathophysiology.

## Abbreviations

The following abbreviations are used in this manuscript:

cAMP	Cyclic adenosine monophosphate
cGMP	Cyclic guanosine monophosphate
COX-2	Inducible cyclooxygenase
EDRF	Endothelium-derived relaxing factor
HBOCs	Haemoglobin-based oxygen carriers
nNO	Nasal NO
NO	Nitric oxide
NOS	Nitric oxide synthase
NOS2	Inducible NOS
NOS3	Endothelial NOS
PCD	Primary ciliary dyskinesia
PDE	Phosphodiesterase
